# Beyond Bloodstream Infection: The Potential for Hierarchical Composite Endpoints in Vascular Access Research

**DOI:** 10.1093/cid/ciag276

**Published:** 2026-04-22

**Authors:** Carl Mellander, Andrew J Martin, Gaurav Langan, Stefanie Seifert, Fredrik Hammarskjöld, Knut Taxbro, Claire M Rickard

**Affiliations:** Department of Biomedical and Clinical Sciences, Linköping University, Linköping, Sweden; Futurum—Academy of Health and Care, Region Jönköping County, Jönköping, Sweden; Department of Anaesthesia and Intensive Care Medicine, Ryhov County Hospital, Jönköping, Sweden; Clinical Trial Capability (ULTRA), The University of Queensland, Brisbane, Queensland, Australia; School of Nursing, Midwifery and Social Work, The University of Queensland, Brisbane, Queensland, Australia; Department of Biomedical and Clinical Sciences, Linköping University, Linköping, Sweden; Department of Anaesthesia and Intensive Care Medicine, Ryhov County Hospital, Jönköping, Sweden; Department of Biomedical and Clinical Sciences, Linköping University, Linköping, Sweden; Department of Anaesthesia and Intensive Care Medicine, Ryhov County Hospital, Jönköping, Sweden; Department of Biomedical and Clinical Sciences, Linköping University, Linköping, Sweden; Department of Anaesthesia and Intensive Care Medicine, Ryhov County Hospital, Jönköping, Sweden; Helicopter Emergency Medical Service, Gothenburg, Sweden; Faculty of Health, Medicine and Behavioural Sciences, The University of Queensland, Brisbane, Queensland, Australia; Herston Infectious Diseases Institute, Metro North Health, Brisbane, Queensland, Australia

To the  Editor—Ong et al provide a valuable and timely explanation of hierarchical composite endpoints (HCEs) and their emerging relevance to infectious diseases research [1]. The authors outline how these endpoints integrate multiple clinical events into a ranked structure that reflects clinical priorities rather than treating all events as equal. This clarification is especially important for clinicians increasingly encountering these methods in modern trials.

We would like to draw attention to the considerable potential for applying HCEs in trials involving vascular access catheters (peripheral, central, venous, arterial) where a variety of infectious and noninfectious complications can be relevant candidates for endpoint construction. Historically, this field has focused primarily on preventing catheter-associated bloodstream infections (CABSIs) or catheter-related deep vein thrombosis [2–4]. Although severe, these events are very uncommon (0.03%–0.08%, peripheral intravenous catheter (PIVC)-CABSI [5], 0.5%–1.4%, central venous catheter (CVC)-CABSI [2]), thus limiting their value as a primary endpoint in clinical trials. In contrast, a range of more frequent complications (31.7%–41.3%, PIVC device failure [5], 12.1%, peripherally inserted central catheter device failure [3]), including insertion failure, occlusion, accidental removal, infiltration, phlebitis, and local infection, exert a heavy cumulative impact on patients, staff, and healthcare systems [5–7]. These complications delay treatment, increase workload, contribute to patient discomfort, and create substantial resource demands, yet they are often underrepresented in outcome measures within vascular access studies [5]. For randomized controlled trials (RCTs) to usefully inform clinical policies and individual decisions about vascular catheter use, a holistic approach to evaluating interventions is needed, which considers the range of potential infectious, mechanical, and patient experiential effects. We would be interested to know whether Ong et al are aware of infectious disease trials in which HCEs have been constructed to incorporate outcomes beyond infection-related endpoints, or if they see other clinical scenarios where this may be valuable.

Restricting outcomes to CABSI alone preserves focus on the most serious adverse catheter event but fails to reflect the broader clinical experience of vascular access. Conversely, combining all complications into a single composite increases event numbers but risks obscuring rare, clinically important events beneath more common, lower-severity problems and may mask opposing effects across components. Furthermore, the HCE example provided by Ong et al (the SCOUT-CAP trial) uses duration of treatment only as a tiebreaker. In that model, antibiotic use was dichotomized (“short” vs “long” categories). In contrast, vascular access outcomes require a more granular time-to-event approach. Because the risk of complication changes daily and catheters are often removed for reasons other than failure (competing risks), HCEs in this field should use exposure-adjusted measures—such as rates per 1000 catheter-days—rather than using time merely as a categorical tiebreaker [8].

The hierarchical structure described by Ong et al provides a pragmatic way to prioritize serious events, such as CABSI, while still accounting for the wider range of complications that shape patient and system outcomes. In other infectious diseases, including nosocomial pneumonia, consensus-based Desirability of Outcome Ranking (DOOR) frameworks have already been developed, demonstrating the value of structured, clinically aligned outcome hierarchies [9]. Developing an analogous DOOR framework for vascular access research would require a systematic approach incorporating consultation with consumer and clinician stakeholders, consensus development, and formal validation processes [10].

Hierarchical composite endpoints should be considered in future trials of catheter interventions, such as antimicrobial locks, dressings, and catheter materials. This could improve the evidence informing catheter selection and infection-prevention by better reflecting clinical impact. We commend the authors for a valuable contribution and hope it encourages further methodological development across infectious diseases and vascular access research.
